# Abbé Hauy

**Published:** 1822-10

**Authors:** 


### OBITUARY.

At Paris, on the 1st of June, the Abbé Hauy, member of the Royal Academy of Sciences, and canon of Notre Dame. This distinguished mineralogist met his death in consequence of a fall, by which he fractured the neck of the os femoris. He fell down on the 14th of May in his cabinet, in consequence, it is believed, of slipping his foot, as he did not subsequently show any affection of the head, which would lead to the idea of his having suffered any apoplectic seizure. He was attended by M. Almand, surgeon to the Salpetriere; but the pain, partly arising from the accident, and partly from an attack of nephralgia, prevented the nature of the accident from being discovered for some time. An abscess formed, which was evacuated; and his strength from this period declined with rapidity, till the morning of the 1st of June, when he died. His interment took place at Père la Chaise, on the 3d of June, which was attended by Gay-Lussac, as president of the Institute, and a considerable body of its members.

